# Early life administration of *Bifidobacterium bifidum BD-1* alleviates long-term colitis by remodeling the gut microbiota and promoting intestinal barrier development

**DOI:** 10.3389/fmicb.2022.916824

**Published:** 2022-07-22

**Authors:** Chenrui Peng, Jinxing Li, Zhonghua Miao, Yunyi Wang, Simou Wu, Yimei Wang, Silu Wang, Ruyue Cheng, Fang He, Xi Shen

**Affiliations:** Department of Nutrition and Food Hygiene, West China School of Public Health and West China Fourth Hospital, Sichuan University, Chengdu, China

**Keywords:** inflammatory bowel disease, early life, *Bifidobacterium bifidum*, gut microbiota, intestinal mucosal barrier, immune response

## Abstract

Inflammatory bowel disease (IBD) is a chronic intestinal disease characterized by microbiota disturbance and intestinal mucosal damage. The current study aimed to investigate the preventive effects of *Bifidobacterium bifidum BD-1* (BD-1) against long-term IBD and possible mechanism by which it alters the gut microbiota, immune response, and mucosal barrier. Our study found that early treatment of BD-1 + Ceftri (ceftriaxone followed by BD-1) and BD-1 confers a certain protective effect against the occurrence of long-term Dextran sulfate sodium-induced colitis, which manifests as a decrease in inflammation scores and MPO activity levels, as well as a relatively intact intestinal epithelial structure. Moreover, compared to BD-1, Ceftri, and NS, early treatment with BD-1 + Ceftri promoted greater expression levels of mucosal barrier-related proteins [KI67, MUC2, ZO-1, secretory immunoglobulin A (slgA), Clauding-1, and Occludin], better local immune responses activation, and moderately better modulation of systemic immune responses during long-term colitis. This may be due to the fact that BD-1 + Ceftri can deliberately prolong the colonization time of some beneficial microbiota (e.g., *Bifidobacterium*) and reduce the relative abundance of inflammation-related microbiota (e.g., *Escherichia/Shigella* and *Ruminococcus*). Interestingly, we found that the changes in the gut barrier and immunity were already present immediately after early intervention with BD-1 + Ceftri, implying that early effects can persist with appropriate intervention. Furthermore, intervention with BD-1 alone in early life confers an anti-inflammatory effect to a certain degree in the long-term, which may be due to the interaction between BD-1 and the host’s native gut microbiota affecting intestinal metabolites. In conclusion, BD-1 was not as effective as BD-1 + Ceftri in early life, perhaps due to its failure to fully play the role of the strain itself under the influence of the host’s complex microbiota. Therefore, further research is needed to explore specific mechanisms for single strain and native microbiota or the combination between probiotics and antibiotics.

## Introduction

Inflammatory bowel disease (IBD) is defined as a chronic, non-specific inflammatory disease of the intestinal tract, mainly including ulcerative colitis and Crohn’s disease. It is characterized by chronic inflammation of the intestinal tract, which predominantly leads to ulceration and erosion (Fan et al., 2021). There may be some key factors in the poorly defined pathogenesis of IBD. Evidence suggests that altered gut microbial composition, immune deficiencies, and impaired barrier function may contribute to the development and progression of IBD (Ananthakrishnan, 2015). In recent years, the prevalence of IBD has been increasing globally, especially in Asia and Latin America. Studies have shown an IBD incidence rate of 1.4 per 100,000 people in Asia and 3.3 per 100,000 in China, with younger patients more likely to develop chronic IBD than adult patients (Kelsen and Baldassano, 2008; Kaplan and Ng, 2017). Thus, IBD has emerged as a global health care burden due to the increasing number of cases and intractability of the disease, suggesting the importance of early prevention and treatment.

Early life is often considered to be one of the critical periods of growth and development, which may be closely associated with adult diseases (e.g., obesity, allergic diseases, and IBD; Arrieta et al., 2014). Early life is also an important stage in the construction of the intestinal microecology. Under the influence of the internal and external environment, the gut microbiota begins in early life and continues to be enriched and adjust in species richness and diversity until it combines with several functions of the intestinal mucosal structure and develops into a mature and stable adult-like intestinal microecology (Jagodzinski et al., 2019; Zhuang et al., 2019). Therefore, promoting or disrupting the process of early gut microbiota construction may have a profound impact on the structure of the long-term microbiota, which can hinder or promote the development of long-term diseases, such as IBD. Our hypothesis is supported by findings suggesting that early exposure to beneficial microbes reduce the risk of IBD (Ng et al., 2015). The mechanisms by which gut microbiota affects IBD disease progression may be closely associated with the intestinal mucosal barrier and host immune function (Kayama et al., 2020). However, there is insufficient evidence and systematic research on whether early changes in the gut microbiota affect the intestinal barrier and immune response to prevent IBD.

In our previous study, we found that the use of *B. bifidum* in early life partially affected the gut microbiota, intestinal epithelial development, and immune indices of mice at day 21, which may have long-term effects. However, the same study showed that the use of ceftriaxone in early life reduced gut microbiota diversity and damaged the crypt structure (Cheng et al., 2019). As such, we hypothesized that the use of antibiotics in early life to destroy the gut microbiota and subsequent use of probiotics to remodel the host microbiota would prevent long-term disease.

Therefore, the current study investigated the mechanisms by which *B. bifidum* in early life improves the gut microbiota, regulates immune response, and promotes intestinal epithelial mucosa development in order to relieve the inflammatory symptoms of long-term chronic colitis. In addition, we further explored the role of probiotics in reconstructing gut microbiota damaged by antibiotics in early life and their effect on long-term Dextran sulfate sodium (DSS)-induced colitis.

## Materials and methods

### Mice

This study utilized 13-day-old timed-pregnant BALB/C female mice (*n* = 35) purchased from Liaoning Changsheng Biotechnology Co., Ltd. and housed in the Animal Center of the West China School of Public Health, Sichuan University (Approval number: SYXK2018-011) under a specific pathogen-free environment. The experimental protocol was approved by the Ethics Committee of West China Fourth Hospital and West China School of Public Health, Sichuan University (Approval number: Gwll2021066).

The average litter size for pregnant BALB/C mice is 6–8 pups. A total of 210 newborn pups were raised in this experiment at an ambient temperature of 23 ± 1°C, 50–70% humidity, and a 12-h light/dark cycle with free access to water and food. Newborn mice were randomly assigned to the NS-water, NS-DSS, Ceftri-water, Ceftri-DSS, BD-1-water, BD-1-DSS, BD-1 + Ceftri-water, and BD-1 + Ceftri-DSS groups.

### Experiment materials

Ceftriaxone (Aladdin Shanghai Biochemical Technology, Shanghai, China) dissolved in saline at 100 mg/kg body weight was used for experimentation. *Bifidobacterium bifidum BD-1* (BD-1; Active bacteria count of 1.25 × 10^11^ CFU/g) was isolated from the intestines of healthy infants in China. The daily intake of probiotics ingested by mice was 10^8^ CFU/g (from 0 to the 7th day) or 10^9^ CFU/g (from the 8th to 21st day). Dextran sulfate sodium (DSS; M.W. 36–50 kDal) was purchased from MP Biomedicals, LLC. (United States).

### Treatment

Mice were treated with saline, ceftriaxone, BD-1, and BD-1 + ceftriaxone (probiotics should be administered 2 h after antibiotics) since birth according to groups. The gavage volume was 10 μl on days 0–7, 100 μl on days 8–14, and 200 μl on days 15–21, after which gavage was stopped. At this time, half of the mice in each group were executed and their organs and feces collected. Mice were provided food and water *ad libitum* until day 42 without any manipulation. On day 42, drinking water in all model groups (DSS groups) was changed to 3% DSS to induce colitis, whereas drinking water in all non-model groups (water groups) remained unchanged. On day 46, the experiment ended and the remaining mice were executed.

### Histopathological analysis

At the end of the experiment, colon tissues were collected from the sacrificed mice, fixed in 10% neutral buffered formalin (Solarbio, Beijing) for 48 h, dehydrated with ethanol, embedded in paraffin, and demolded after freezing at −18°C. Subsequently, each sample was sectioned and stained with hematoxylin and eosin. Finally, the sample sections were observed under a light microscope (Olympus, Tokyo, Japan), and the pathological condition of colon tissues was evaluated by a professional pathology teacher.

Inflammatory pathology in colon tissues was scored according to the following criteria: inflammation (grade 0, none; grade 1, slight; grade 2, moderate; and grade 3, severe); crypt damage (grade 0, intact crypts; grade 1, loss of the bottom third; grade 2, loss of the bottom two thirds; grade 3, loss of the entire crypt with surface epithelium intact; and grade 4, loss of the entire crypt and surface epithelium); percentage of area involved according to inflammation (grade 1, 1–25%; grade 2, 26–50%; grade 3, 51–75%; and grade 4, 76–100%); percentage of crypt damage (grade 1, 1–25%; grade 2, 26–50%; grade 3, 51–75%; and grade 4, 76–100%); and depth of inflammation (grade 0, normal; grade 1, mucosa; grade 2, submucosa; and grade 3, transmural). The final pathology score of a colonic tissue was the sum of the component scores (Zhu et al., 2015; Sang et al., 2016; Ding et al., 2019).

### Colonic myeloperoxidase activity level assays

Colon tissues (20 mg) were homogenized in 150 μl phosphate buffer saline (PBS) and then centrifuged at 2000 × *g* for 10 min. Next, the sample supernatant was collected. Measurements were conducted according to the manufacturer’s protocol of the ab275109 Mouse Myeloperoxidase (MPO) SimpleStep ELISA® Kit (Abcam, Shanghai, China). During the whole experiment, the absorbance at 600 or 450 nm was recorded using a microplate reader (Thermo Fisher, Shanghai, China).

### 16S rRNA sequence

Fresh feces from mice were collected on day 21 and 46 and subsequently frozen at −80°C. Fecal genomic DNA was extracted from mice feces (200 mg) at each time point following the manufacturer’s instructions for the TIANamp Stool DNA Kit (Tiangen, Beijing, China). The V3–V4 regions of bacterial 16S rRNA were amplified using primers 341 F: 5′-actcctacgggrsgcagag-3′ and 806 R: 5′-GGACTACVV GGGTATCTAATC-3′ using PCR to generate the sequencing library. All PCR reactions in this process were performed using 15 μl Phusion High-Fidelity PCR Master Mix (New England Biolabs). The PCR reaction procedure were as follows: initial denaturation step at 98°C for 1 min, followed by 30 cycles (denaturation at 98°C for 10 s, annealing at 50°C for 30 s, and extension at 72°C for 30 s), and finally at 72°C for 5 min. Subsequently, PCR products were purified with QIAquick Gel Extraction Kit (Qiagen, Germany). Finally, the library was generated by instructions for TruSeq DNA PCR-Free Sample Prep kit (Illumina, America) and sequenced on the Illumina NovaSeq platform. After data preprocessing, the valid sequences are clustered to form operational taxonomic units (OTUs), which were annotated by the RDP databased. Then alpha diversity indexes (ACE, Chao1, Simpson, Shannon, etc.) and beta diversity indexes (Bray_Cutis, UniFrac, etc.) were calculated by Phyloseq Package in R software. The Simpson index is obtained after log10 transformation.

The EDDA Package in R software was used to analyze the differences in OTUs abundance on different taxonomic levels of classification using Metastats, and *p* < 0.05 was considered significant difference. One-way ANOVA or Kruskal-Wallis tests were used to evaluate changes in alpha diversity indexes. For beta diversity analysis, the principal coordinate analysis (PCoA) was performed based on the weighted UniFrac distance and permutational multivariate ANOVA (PERMANOVA) was used to calculate significant differences in microbial composition between groups with “adonis” (PERMANOVA) and “betadisper” (multivariate homogeneity of group dispersions) tests.

### Short-chain fatty acid analysis

Mice feces (100 mg) were homogenized using 100 μl 15% phosphoric acid, 100 μl 50 μg/ml of internal standard (isocaproic acid), and 400 μl diethyl ether for 1 min and then centrifuged at 12,000 rpm for 10 min at 4°C. The supernatant was then obtained and measured on the Agilent 7890B gas chromatograph (Agilent, Santa Clara, CA, United States). The following short-chain fatty acids (SCFAs) were included in the assay: acetic acid, propionic acid, butyric acid, isobutyric acid, valeric acid, isovaleric acid, and caproic acid. The chromatographic conditions were as follows: Agilent HP-INNOWAX capillary column (Agilent, China), split injection, injection volume 1 μl, and split ratio 10:1. The inlet temperature was set as follows: inlet, 250°C; transfer line, 250°C; ion source, 230°C; and the quadrupole, 150°C.

### Secretory immunoglobulin A level assays

The cecal contents (50 mg) were soaked in 200 μl PBS, homogenized, and then centrifuged at 1,000 × *g* for 10 min at room temperature. The supernatant was used for further analysis. The sample supernatant was diluted 2,500 times on day 21 and diluted 10,000 times on day 46. The assay was performed according to the instructions for the Mouse sIgA (Secretory Immunoglobulin A) ELISA Kit (Elabscience Biotechnology Co., Ltd., Wuhan, China). Finally, the absorbance at 450 nm was recorded using a microplate reader (Thermo Fisher, Shanghai, China). The data were fit with a four-parameter logistic function.

### Serum cytokines levels analysis

Blood from mice was collected at days 21 and 46 using ocular blood sampling and centrifuged at 2,000 *g* for 15–20 min at 4°C. The supernatant obtained in the previous experiment was then centrifuged at 2,000 *g* for 5 min at 4°C to obtain the sample to be tested. The concentrations of TNF-α, IL-6, IL-10, IL-1β, IL-12(P70), and IFN-γ in the serum samples were analyzed using a Mouse Magnetic Luminex® Assays (Bio-Techne Corporation, United States) and measured using a Luminex 200TM multiplexing instrument (Merck Millipore, United States). None of the samples were diluted.

### Splenic and colonic cytokines mRNA expression

The colon and spleen were weighed separately (10–15 mg) into a 2 ml Lysing Matrix D tube (MP Biomedicals, United States) at days 21 and 46. The tissue was added 500 μl Buffer RL1 (Foregene, Chengdu, China) and then homogenized using the FastPrep-24 (MP Biomedicals, United States) homogenizer for 20 s at 4 m/s. Total RNA was extracted from the tissues according to the manufacturer’s instructions for the Animal Total RNA Isolation Kit (Foregene, Chengdu, China). Reverse transcription of total RNA into cDNA was achieved using iScriptTM cDNA Synthesis Kit (Bio-Rad, Hercules, CA, United States) in a C1000 Touch Thermal Cycler (Bio-Rad). The RT-PCR reaction process was as follow: 25°C for 5 min, followed by 46°C for 20 min, and finally 95°C for 1 min. Quantitative real-time PCR of cDNA was then performed on a CFX96 system (Bio-Rad) using a SsoFastTM EvaGreen® Supermix (Bio-Rad). The QPCR cycling conditions were as follows: reaction with an initial denaturation step at 98°C for 30 s, followed by 39 cycles of denaturation at 98°C for 15 s, and annealing temperature with an extension step for 30 s at 60°C. Splenic and colonic cytokines mRNA expression levels were normalized against β-actin.

Throughout the current study, the concentrations of KI67, MUC2, ZO-1, Clauding-1, and Occludin in colon samples and TNF-α, IL-6, IL-10, IL-12 (P40), and IL-17a in spleen and colon samples were analyzed. All primers were synthesized by Sangon Biotech (Shanghai, China; Table 1).

**Table 1:** Primers sequences for RT-PCR.

Gene	Primers
β-actin-F	5′-GTGGGCCGCTCTAGGCACCAA-3′
β-actin-R	5′-CTCTTTGATGTCACGCACGATTTC-3′
IL-6-F	5′-GTCACAGAAGGAGTGGCTA-3′
IL-6-R	5′-AGAGAACAACATAAGTCAGATACC-3′
IL-10-F	5′-GACCAGCTGGACAACATACT-3′
IL-10-R	5′-GAGGGTCTTCAGCTTCTCAC-3′
IL-12P40-F	5′-CTCTGTCTGCAGAGAAGGTC-3′
IL-12P40-R	5′-GCTGGTGCTGTAGTTCTCAT-3′
TNF-α-F	5′-CTCTTCAAGGGACAAGGCTG-3′
TNF-α-R	5′-CGGACTCCGCAAAGTCTAAG-3′
IL-17a-F	5′-TGATGCTGTTGCTGCTGCTGAG-3′
IL-17a-R	5′-CACATTCTGGAGGAAGTCCTTGGC-3′
Claudin-1-F	5′-GCTGGGTTTCATCCTGGCTTCTC-3′
Claudin-1-R	5′-CCTGAGCGGTCACGATGTTGTC-3′
Occludin-F	5′-GCGAGGAGCTGGAGGAGGAC-3′
Occludin-R	5′-CGTCGTCTAGTTCTGCCTGTAAGC-3′
ZO-1-F	5′-GCGAACAGAAGGAGCGAGAAGAG-3′
ZO-1-R	5′-GCTTTGCGGGCTGACTGGAG-3′
KI67-F	5′-GCCTGCCCGACCCTACAAAATG-3′
KI67-R	5′-CTCATCTGCTGCTGCTTCTCCTTC-3′
MUC2-F	5′-TGCTGACGAGTGGTTGGTGAATG-3′
MUC2-R	5′-TGATGAGGTGGCAGACAGGAGAC-3′

### Statistical analysis

The data analysis was performed using SPSS 26.0 software (SPSS Inc., Chicago, IL). Data were presented as means ± SDs. One-way ANOVA or Kruskal–Wallis H-test was used for multiple comparisons, and *Post hoc* pairwise comparisons were performed using LSD and Bonferroni tests to adjust for multiple comparisons. *p* < 0.05 was considered to indicate a statistically significant difference. All statistical tests were two-tailed tests.

## Results

### Colonic inflammation after DSS induction at day 46

In this experiment, mice were randomly assigned to the NS-water, NS-DSS, Ceftri-water, Ceftri-DSS, BD-1-water, BD-1-DSS, BD-1 + Ceftri-water, and BD-1 + Ceftri-DSS groups from birth according to the time and type of intervention in early life. All treatments conducted in this study were stopped at day 21, and after a period of time, colitis was induced with DSS from days 42 to 46.

**Figure 1:**
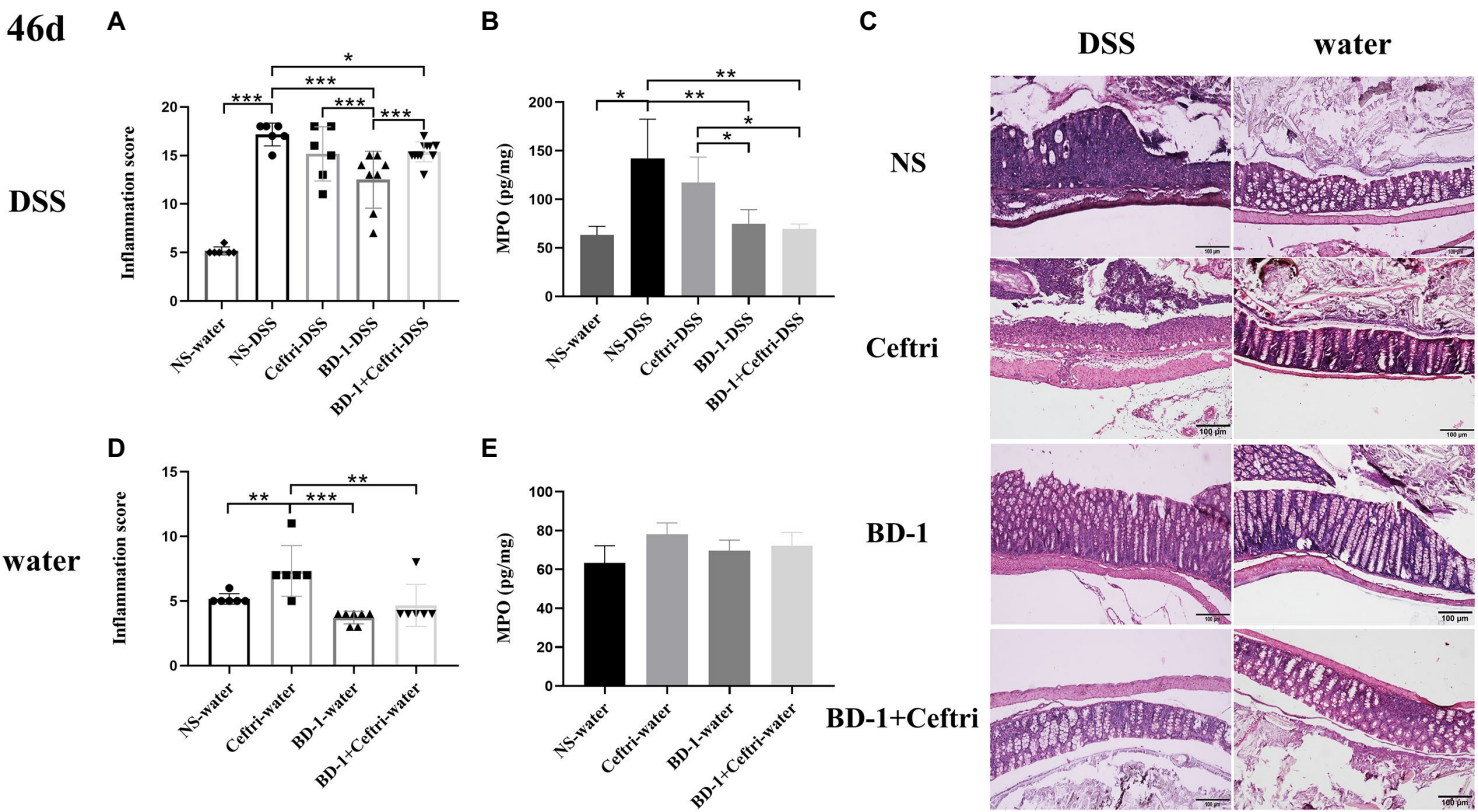
Colonic inflammation after Dextran sulfate sodium (DSS) induction (46 days, *n* = 6). **(A)** Inflammatory pathology score (DSS groups). **(B)** Myeloperoxidase activity (DSS groups). **(C)** Typical histological images of H&E-stained colonic tissue in NS-DSS group, NS-water group, Ceftri-DSS group, Ceftri-water group, BD-1-DSS group, BD-1-water group, BD-1 + Ceftri-DSS group, and BD-1 + Ceftri-water group. Microscope magnification: 10x and 10x. **(D)** Inflammatory pathology score (water groups). **(E)** Myeloperoxidase activity (water groups). ^*^p < 0.05, ^**^p < 0.01, and ^***^p < 0.001 as conducted.

At the end of the experiment at day 46, the NS-DSS group had significantly higher inflammatory scores and MPO activity level (*p* < 0.001 and *p* < 0.05, respectively; Figures 1A,B), as well as more severe inflammatory infiltration and crypt damage, compared to the NS-water group (Figure 1C). As shown in Figure 1, the BD-1-DSS group and BD-1 + Ceftri-DSS group showed significantly lower inflammatory scores and MPO activity levels (*p* < 0.001 and *p* < 0.01, respectively), as well as milder inflammatory damage, compared to the NS-DSS group. Meanwhile, compared to the Ceftri-DSS group, the BD-1 + Ceftri-DSS group had a significantly lower MPO activity level and more intact colonic mucosal epithelial cell structure (*p* < 0.05). Similarly, among the non-inflammatory groups, the BD-1-water and BD-1 + Ceftri-water groups also showed lower inflammatory states compared to the rest of the groups (Figures 1C,D). The difference in MPO activity was not statistically significant in the non-inflammatory groups (*p* > 0.05, Figure 1E).

### Changes in the colonic mucosal barrier after long-term colitis at day 46

**Figure 2:**
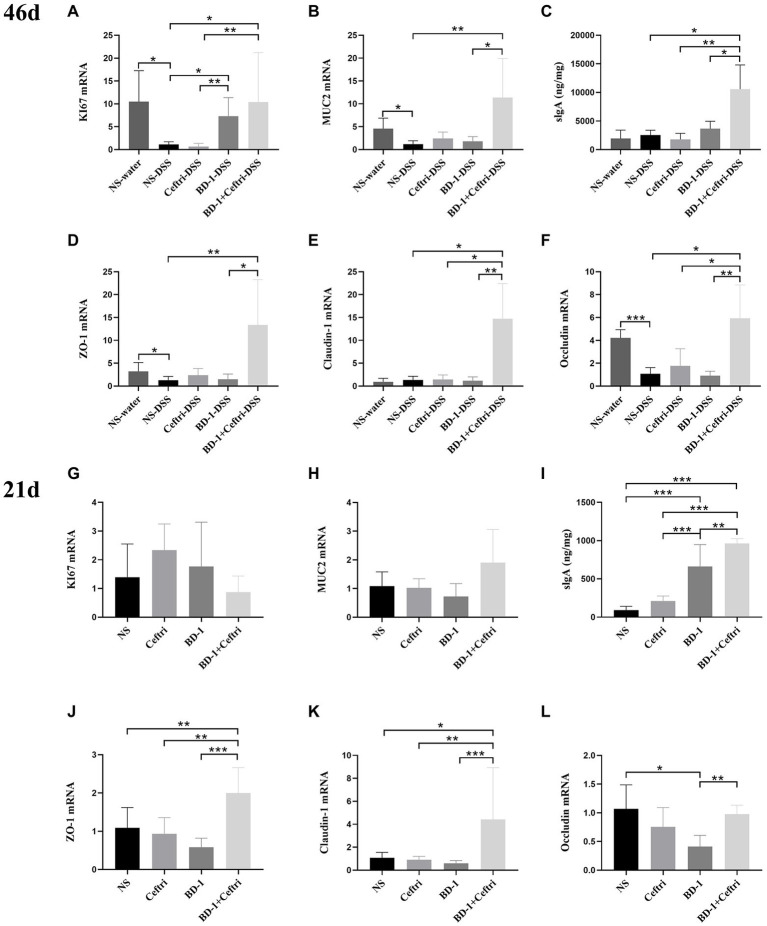
Colonic mucosal barrier condition after DSS induction and early life intervention (46 and 21 days, respectively; *n* = 6). **(A–F)** on day 46 and **(G–L)** on day 21. **(A)** Colonic KI67 mRNA level. **(B)** Colonic MUC2 mRNA level. **(C)** Secretory Immunoglobulin A (sIgA) level in the cecum faeces. **(D)** Colonic ZO-1 mRNA level. **(E)** Colonic Claudin-1 mRNA level. **(F)** Colonic Occludin mRNA level. **(G)** KI67 mRNA level. **(H)** MUC2 mRNA level. **(I)** SIgA level in the cecum faeces. **(J)** ZO-1 mRNA level. **(K)** Claudin-1 mRNA level. **(L)** Occludin mRNA level. ^*^p < 0.05, ^**^p < 0.01, and ^***^p < 0.001 as conducted.

As shown in Figure 2, the NS-DSS group had a significantly lower expression of KI67 mRNA, MUC2 mRNA, ZO-1 mRNA, and Occludin mRNA after DSS induction compared to the NS-water group (*p* < 0.05, respectively). In terms of proliferation changes (Figure 2A), the BD-1-DSS and BD-1 + Ceftri-DSS groups had significantly higher KI67 mRNA expression compared to the NS-DSS and Ceftri-DSS groups (*p* < 0.05, all). In particular, KI67 mRNA expression in the BD-1 + Ceftri-water group was highest among the non-inflammatory groups (*p* < 0.05; Supplementary Figure S1A). In terms of mucosal barrier changes (Figures 2B,C), the BD-1 + Ceftri-DSS group had significantly higher MUC2 mRNA expression and slgA levels compared to the NS-DSS group (*p* < 0.01 and *p* < 0.05, respectively). In the case of colonic mechanical barrier changes (Figures 2D–F), the BD-1 + Ceftri-DSS group had significantly higher ZO-1 mRNA expression than the NS-DSS group and had the highest Claudin-1 mRNA and Occludin mRNA levels among the inflammatory groups (*p* < 0.05, all). Changes in MUC2 mRNA, slgA, Claudin-1 mRNA, and Occludin mRNA can be seen in the Supplementary material (Supplementary Figures S1A–H).

### Intestinal development after treatment at day 21

The mRNA expression of KI67 mRNA and MUC2 did not significantly differ between the groups (*p* > 0.05; Figures 2G–H). The BD-1 and BD-1 + Ceftri groups had significantly higher slgA levels compared to the NS and Ceftri groups, with the BD-1 + Ceftri group having significantly higher slgA levels than the BD-1 group (*p* < 0.01, all, respectively; Figure 2I). ZO-1 mRNA and Clauding-1 mRNA expression were higher in the BD-1 + Ceftri group than in the NS, Ceftri, and BD-1 groups (*p* < 0.05, all, respectively; Figures 2J,K). Occludin mRNA expression was higher in the BD-1 + Ceftri group than the BD-1 group (*p* < 0.05; Figure 2L).

From the results of the intestinal mucosal barrier on days 46 and 21, early intervention with BD-1 reduces long-term inflammatory symptoms in the colon, which did not affect mucus or stem cell proliferation and differentiation on day 21 but increased KI67 mRNA expression on day 46. Surprisingly, early treatment with BD-1 + Ceftri had long-term anti-inflammatory effect, enhanced mucosal immunity function, and improved tight junction expression until the long term.

### Colonic local and systemic immune status at day 46

**Figure 3:**
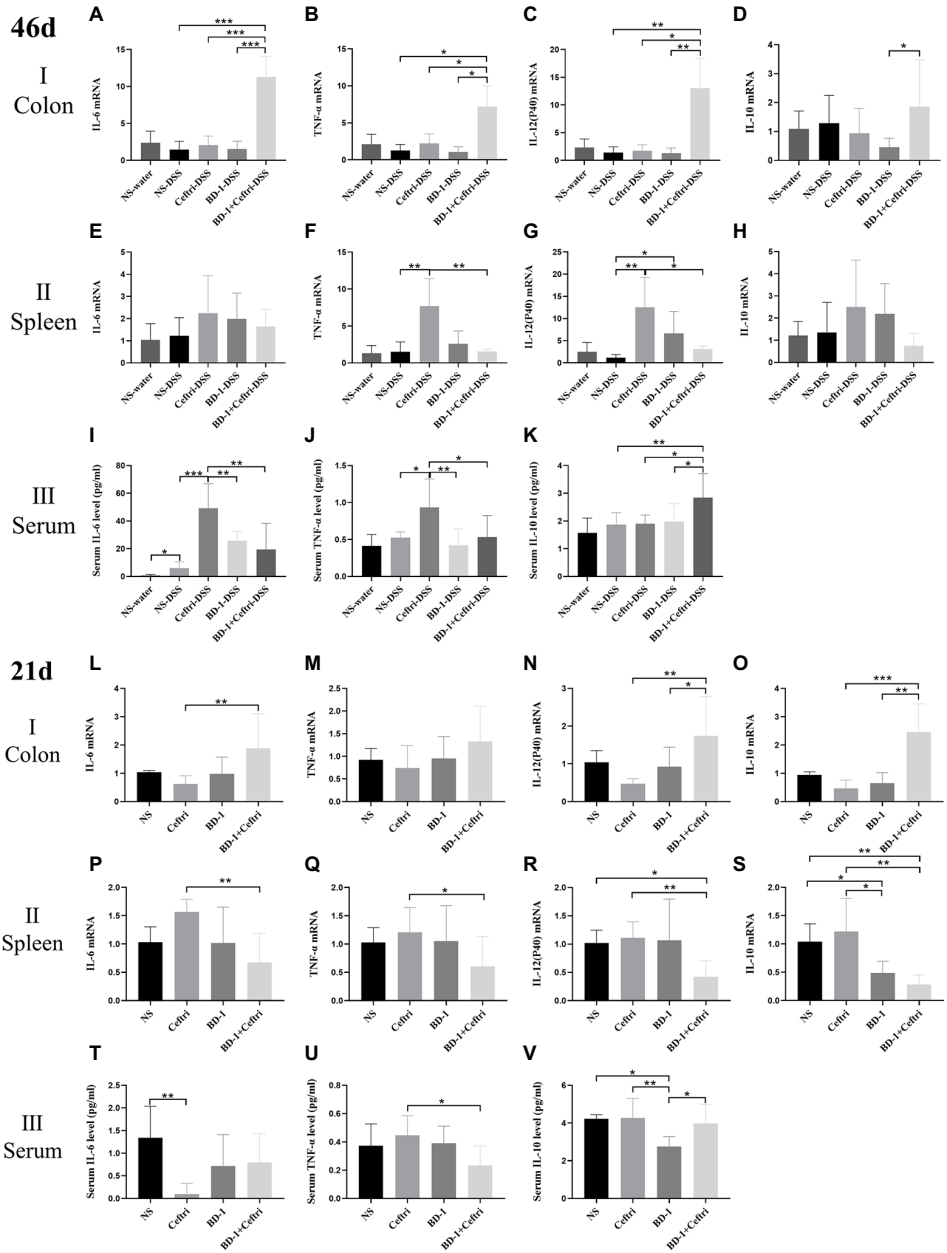
Local and systemic immunity after DSS induction and early life intervention (46 and 21 days, respectively; *n* = 6). **(A–K)** on day 46 and **(L–V)** on day 21. **(A)** Colonic IL-6 mRNA level. **(B)** Colonic TNF-α mRNA level. **(C)** Colonic IL-12 (P40) mRNA level. **(D)** Colonic IL-10 mRNA level. **(E)** Splenic IL-6 mRNA level. **(F)** Splenic TNF-α mRNA level. **(G)** Splenic IL-12(P40) mRNA level. **(H)** Splenic IL-10 mRNA level. **(I)** Serum IL-6 level. **(J)** Serum TNF-α level. **(K)** Serum IL-10 level. **(L)** Colonic IL-6 mRNA level. **(M)** Colonic TNF-α mRNA level. **(N)** Colonic IL-12(P40) mRNA level. **(O)** Colonic IL-10 mRNA level. **(P)** Splenic IL-6 mRNA level. **(Q)** Splenic TNF-α mRNA level. **(R)** Splenic IL-12 (P40) mRNA level. **(S)** Splenic IL-10 mRNA level. **(D)** Serum IL-6 level. **(U)** Serum TNF-α level. **(V)** Serum IL-10 level. ^*^p < 0.05, ^**^p < 0.01, and ^***^p < 0.001 as conducted.

Regarding colonic immune changes (Figures 3A–D), the BD-1 + Ceftri-DSS group had the highest expression of IL-6 mRNA, TNF-α mRNA, and IL-12(P40) mRNA among the inflammation groups (*p* < 0.05, all) and had a higher IL-10 mRNA expression compared to the BD-1-DSS group (*p* < 0.05). Changes in colonic IL6 mRNA, TNF-α mRNA, IL-12 (P40) mRNA, and IL-10 mRNA can be seen between the Supplementary materials (Supplementary Figures S2A–D). No significant difference in IL-17a mRNA expression was observed among all groups (*p* > 0.05; Supplementary Figures S2E,F). Regarding systemic immune changes (Figures 3E–K), the Ceftri-DSS group had significantly higher splenic TNF-α mRNA, splenic IL-12 (P40) mRNA, serum IL-6, and TNF-α levels than the NS-DSS and BD-1 + Ceftri-DSS groups (*p* < 0.05), whereas no significant differences were found between the NS-DSS and BD-1 + Ceftri-DSS groups (*p* > 0.05). Moreover, serum IL-10 levels were highest in the BD-1 + Ceftri-DSS group (*p* < 0.05). Changes in splenic IL6 mRNA, TNF-α mRNA, IL-12 (P40) mRNA, and IL-10 mRNA can be seen between the Supplementary material (Supplementary Figures S2G–J). No significant differences between in splenic IL-6 mRNA, IL-10 mRNA, and IL-17a mRNA expression were observed among the DSS groups (*p* > 0.05; Figures 3E,H and Supplementary Figure S2K, respectively); similarly, no significant differences in splenic IL-17a mRNA expression, serum IL-6, TNF-α, and IL-10 levels were found among the water groups (*p* > 0.05; Supplementary Figures S2L–O). Serum IL-1β, IL-12(P70), and IFN-γ levels were below the limit of detection.

### Colonic and systemic immune response at day 21

Regarding the colonic local immune response (Figures 3L–O), the BD-1 + Ceftri group had a significantly higher IL-6 mRNA, IL-12 (P40) mRNA, and IL-10 mRNA expression compared to the Ceftri group (*p* < 0.01, all), with no difference in TNF-α mRNA expression between the BD-1 + Ceftri and Ceftri groups (*p* > 0.05). Regarding systemic immune response (Figures 3P–V), the BD-1 + Ceftri group had lower splenic IL-6 mRNA, TNF-α mRNA, IL-12(P40) mRNA, IL-10 mRNA, and serum TNF-α levels compared to the Ceftri group (*p* < 0.05, all), with no significant difference in serum IL-6 and IL-10 levels between the two groups (*p* > 0.05, both).

Combining the results of colonic and systemic immunity on days 46 and 21, we found that compared with Ceftri and BD-1 separately, early treatment with BD-1 + Ceftri was more able to stimulate local immunity and effectively reduce the levels of systemic inflammation on days 46 and 21, but the aforementioned effects were generally stronger on day 46.

### Changes in gut microbiota under the influence of forward colitis at day 46

**Figure 4:**
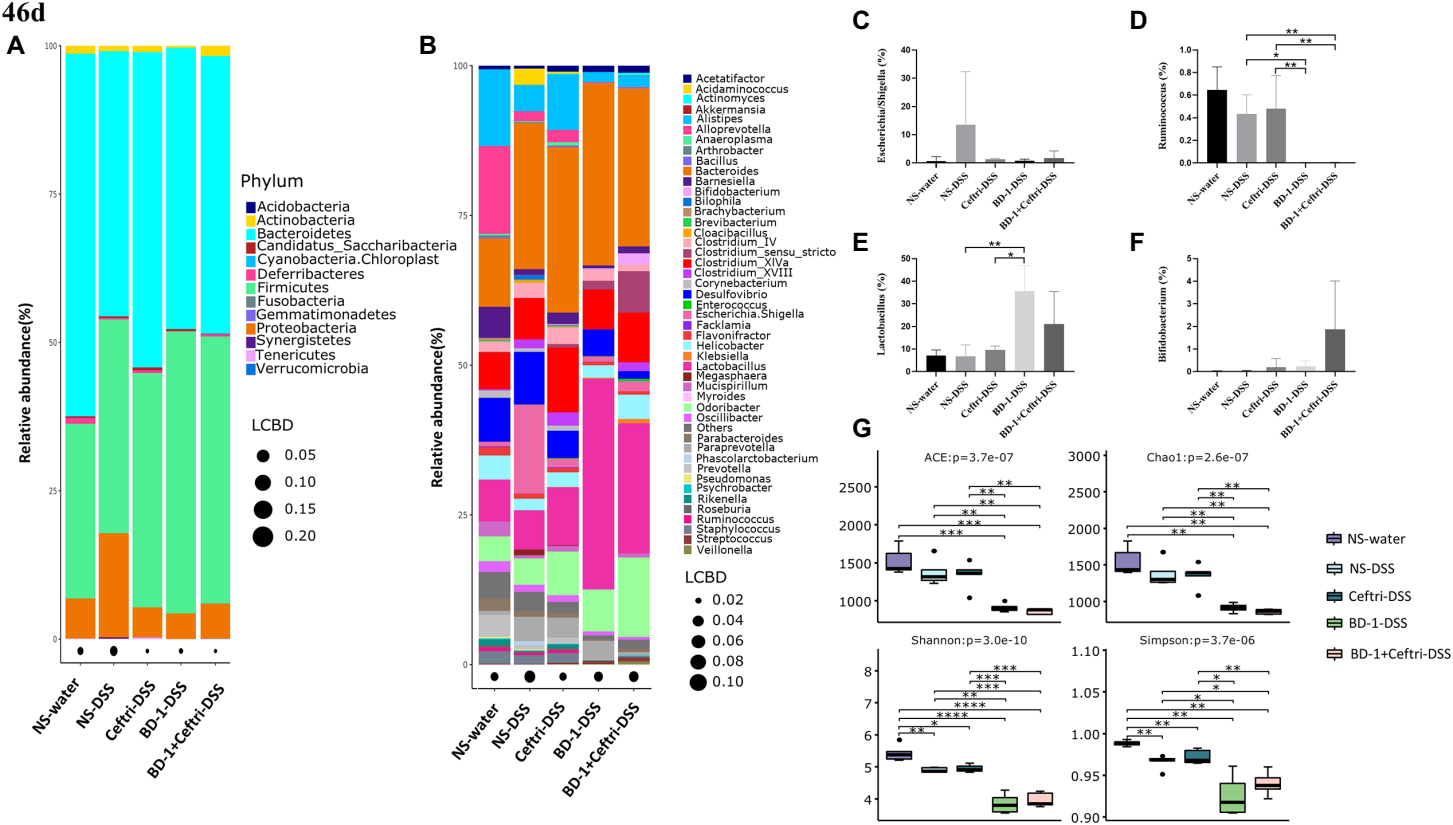
Changes in gut microbiota and metabolites after DSS induction (46 days, *n* = 5). **(A)** Relative abundance at the phylum level. **(B)** Relative abundance at the genus level. **(C)** Relative abundance of *Escherichia/Shigella*. **(D)** Relative abundance of *Ruminococcus*. **(E)** Relative abundance of *Lactobacillus*. **(F)** Relative abundance of *Bifidobacterium*. **(G)** The alpha diversity of the gut microbiota. ^*^p < 0.05, ^**^p < 0.01, and ^***^p < 0.001 as conducted.

After experimentation, the composition of the gut microbiota was analyzed. At the phylum level (Figure 4A), the NS-DSS group showed a lower relative abundance of *Bacteroidetes* (44.68 vs. 61.13%) and a higher relative abundance of *Firmicutes* (36.02 vs. 29.46%) and *Proteobacteria* (17.53 vs. 6.75%) compared to the NS-water group. The NS-DSS group had a greater relative abundance of *Proteobacteria* compared to the Ceftri-DSS, BD-1-DSS, and BD-1 + Ceftri-DSS groups (17.53 vs. 5.02%, 4.33 and 5.91%, respectively). At the genus level (Figure 4B), the NS-DSS group had a higher relative abundance of *Escherichia/Shigella* compared to the NS-water group (14.82 vs. 0.75%). Moreover, the NS-DSS group had a greater relative abundance of *Escherichia/Shigella* (14.82 vs. 0.84% and 1.68%, respectively) but a significantly greater relative abundance of *Ruminococcus* compared to the BD-1-DSS and BD-1 + Ceftri-DSS groups (*p* < 0.05, both; Figures 4C,D). Similarly, compared to the NS-DSS group, the BD-1-DSS group had significantly higher relative abundance of *Lactobacillus* (*p* < 0.05), whereas the BD-1 + Ceftri-DSS group had a higher relative abundance of *Bifidobacterium* (0.02 vs. 1.86%, Figures 4E,F). The aforementioned trends were also observed in the non-inflammatory groups (Supplementary Figures S3A–G). In terms of alpha diversity, the BD-1-DSS and BD-1 + Ceftri-DSS groups had lower ACE, Chao1, and Shannon and Simpson metrics compared to the NS-DSS and Ceftri-DSS groups (*p* < 0.01, all; Figure 4G). On day 42, compared with the NS and Ceftri groups, there were still significant differences in the phylum level, genus level, alpha diversity, and beta diversity of gut microbiota in BD-1 and BD-1 + Ceftri groups after early intervention (Supplementary Figures S4A–D).

For intestinal metabolites, no significant differences in the concentrations of acetic acid, propionic acid, and butyric acid were observed between the NS-DSS group and other groups (*p* > 0.05, all; Supplementary Figures S5A–C). However, compared to the NS-water group, BD-1-water and BD-1 + Ceftri-water groups had significantly greater concentrations of acetic acid and butyric acid (*p* < 0.05, respectively; Supplementary Figures S5D–F).

### Changes in gut microbiota and metabolites after treatment at day 21

**Figure 5:**
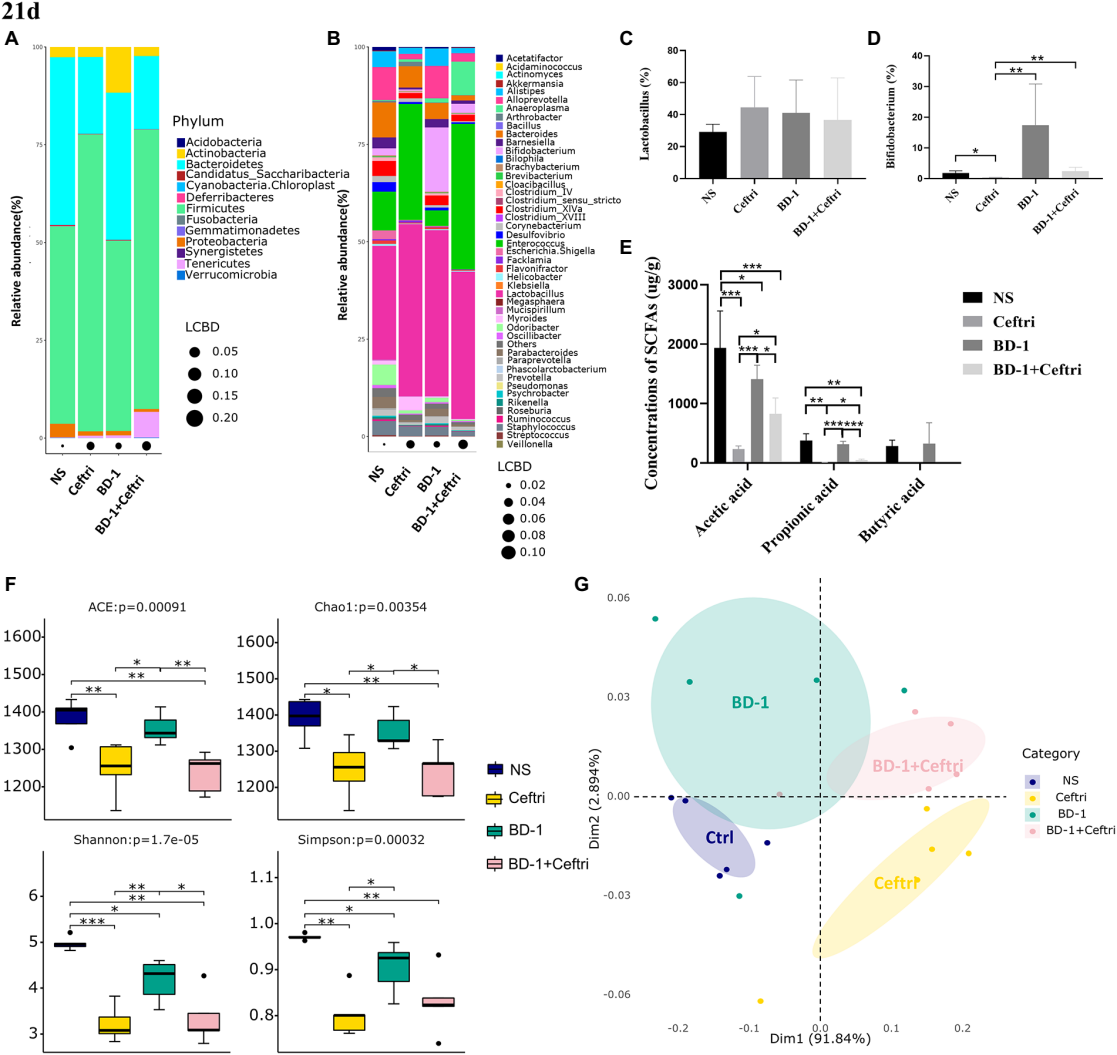
Effects on gut microbiota and metabolites after early life intervention (21 days). **(A)** Relative abundance at the phylum level (*n* = 5). **(B)** Relative abundance at the genus level (*n* = 5). **(C)** Relative abundance of *Lactobacillus* (*n* = 5). **(D)** Relative abundance of *Bifidobacterium* (*n* = 5). **(E)** Concentrations of SCFAs (Acetic acid, Propionic acid, and Butyric acid, respectively; *n* = 6). **(F)** The alpha diversity of the gut microbiota (*n* = 5). **(G)** Principal co-ordinates analysis (PCoA) plots of fecal microbiota (Adnois, *p* < 0.05; Betadisper, *p* > 0.05; *n* = 5). ^*^p < 0.05, ^**^p < 0.01, and ^***^p < 0.001 as conducted.

Experimental treatment was started since birth until day 21, after which the gut microbiota in the faces was systematically analyzed. At the phylum level (Figure 5A), the Ceftri and BD-1 + Ceftri groups had a higher relative abundance of *Firmicutes* and a lower relative abundance of *Bacteroidetes* compared to the other groups (*Firmicutes*: 75.92 and 71.40%, *Bacteroidetes*: 19.67 and 18.73%, respectively). Furthermore, among the groups, the BD-1 group had the highest relative abundance of *Actinobacteria*, whereas the BD-1 + Ceftri group had the lowest relative abundance of *Proteobacteria* but the highest relative abundance of *Tenericutes* (11.71, 0.72, and 6.61%, respectively; Figure 5A). At the genus level (Figure 5B), the Ceftri group had the lowest relative abundance of *Alistipeshe*, *Clostridium_XlVa*, and *Bifidobacterium* and the highest relative abundance of *Enterococcus* (1.58, 1.37, 0.37, and 29.77%, respectively). The relative abundance of *Lactobacillus* did not differ significantly between these groups, whereas the relative abundance of *Bifidobacterium* was specifically higher in the BD-1 group (*p* > 0.05 and 16.48%, respectively; Figures 5C,D).

As shown in Figure 5E, both the Ceftri and BD-1 + Ceftri groups had significantly lower acetic and propionic acid concentrations compared to the NS and BD-1 groups, but the BD-1 + Ceftri group had higher acetic and propionic acid concentrations compared to the Ceftri group (*p* < 0.05, all). Butyric acid content was below the detection limit in the Ceftri and Ceftri + BD-1 groups and did not differ between the NS and BD-1 groups.

In terms of alpha diversity, compared to the NS group, both the Ceftri and BD-1 + Ceftri groups had significantly lower ACE, Chao1, Shannon and Simpson metrics, and the BD-1 group had significantly lower Shannon and Simpson metrics (*p* < 0.05, all; Figure 5F). Similarly, compare to the BD-1 group, the Ceftri group had significantly lower ACE, Chao1, Shannon and Simpson metrics, and the BD-1 + Ceftri group had significant lower ACE, Chao1, and Shannon metrics (*p* < 0.05, all; Figure 5F). In beta diversity analysis (Figure 5G), significant differences in the structure of the microbial community were observed between the NS and BD-1 groups based on the results of the weighted unifrac metric. The microbial communities of the Ceftri and BD-1 + Ceftri groups are in different quadrants and can be separated under principal coordinates analysis (*p* < 0.05).

For the changes in gut microbiota on days 46 and 21, we believe that early intervention with BD-1 increased the relative abundance of beneficial bacteria such as *Bifidobacterium* and produce short-chain fatty acids, but the early probiotic effects did not persist into the long term. However, compared with BD-1 separately, early intervention with BD-1 + Ceftri prolongs the survival time of certain bacteria (e.g., *Bifidobacterium*) and effectively influenced the relative abundance of inflammation-related microbiota (e.g., *Escherichia/Shigella* and *Ruminococcus*).

## Discussion

Recently, there has been a growing concern regarding the early years of life, particularly in terms of the potential for early life to be a critical window for the development of several diseases, including IBD. Early breastfeeding, delivery practices, and antibiotic exposure have been suggested as possible potential risk factors for IBD, and dysbiosis of intestinal microecology has been closely associated with the development and progression of IBD (Ananthakrishnan et al., 2018; Lee and Chang, 2021). As such, specific improvements in the gut microbiota might open new methods for the prevention and treatment of IBD (Glassner et al., 2020). Endt et al. pointed out that when an infection occurs in the host’s gut, the body protects itself from disease by adjusting the normal microbiota to rebuild and gradually clear the infectious pathogen (Endt et al., 2010). Therefore, the current study aimed to determine the immediate and lasting effects of probiotic and antibiotic exposure in early life on gut microbiota and on DSS-induced colitis severity. Simultaneously, we used probiotics to specifically influence intestinal microecology, based on the destruction of bacterial community structure by antibiotics in early life referring to the principle of fecal microbiota transplantation (FMT; Weingarden and Vaughn, 2017). This could be a potential strategy for preventing IBD.

At the end of the trial on day 46, we comprehensively analyzed inflammation scores, typical pathology section images, and MPO activity levels to determine the level of inflammation in DSS-induced colitis. Increased MPO activity has been associated with colitis severity and can be used as a biomarker of inflammation (Kim et al., 2012; Hansberry et al., 2017). After DSS induction, the NS-DSS group showed significantly greater inflammation scores, MPO levels, and crypt damage severity. The aforementioned results suggest that the DSS-induced colitis model was successful. Obviously, the use of BD-1 and BD-1 + Ceftri in early life reduced inflammation scores and MPO activity levels, relieved inflammatory symptoms, and partially protected colonic mucosal structures when long-term colitis occurred. Simultaneously, they also promoted lower inflammation scores and more intact crypt structure in the non-inflammatory groups. Therefore, our results suggested that the use of BD-1 and BD-1 + Ceftri in early life both prevent intestinal inflammatory damage caused by long-term DSS after stopping the intervention, which effectively alleviates inflammatory symptoms and reduces inflammatory factors.

Studies have shown that intestinal epithelial barrier dysfunction and impaired tight junction function play a crucial role in pathogenesis of IBD (Sanchez de Medina et al., 2014; Mehandru and Colombel, 2021). Cell proliferation and the expression of tight junction-associated and peripheral membrane proteins (KI67, Occluding, Claudin-1, ZO-1, and MUC2) have been analyzed to determine intestinal barrier function (Tan and Zheng, 2018; Graefe et al., 2019; Yao et al., 2021). The slgA is the intestinal mucosal immune barrier’s front line against external pathogens (Kumar et al., 2020). The current study found that at day 46, DSS-induced colitis resulted in a significant decrease in KI67, MUC2, ZO-1, and Occluding mRNA expression in the colon tissues. However, the BD-1 + Ceftri-DSS group showed a specific upward trend in KI67, MUC2, slgA, ZO-1, Claudin-1, and Occludin mRNA expression in the inflammatory groups. Similar findings have been obtained in the experiment results of Tan and Zheng (2018) (Hou et al., 2020; Wu et al., 2021). Moreover, trends similar to those described above have emerged on day 21. Clearly, early treatment with BD-1 + Ceftri can better increase expression of slgA, ZO-1, and Claudin-1 mRNA expression than NS, Ceftri, and BD-1, suggesting the apparent benefit of BD-1 + Ceftri for promoting intestinal development. Our findings showed the effect of appropriate intervention in early life can last until the occurrence of long-term colitis. Accordingly, we found that treatment with a single strain in early life could alleviate inflammatory symptoms after long-term DSS induction but did not directly reverse gut barrier damaged after inflammation. In contrast, treatment with a single strain after using antibiotics in early life showed significant intestinal barrier repair during long-term colitis. Therefore, our findings highlight the direct restoration of intestinal barrier by a single strain could under the condition of inhibiting the intestinal proto-microbiota.

To further explore the effects and mechanisms of the strain, the current study determined immune-related indexes. Innate and adaptive immune dysfunction has been showed to facilitate the development of abnormal intestinal inflammatory symptoms in patients with IBD, with research focusing on pro- and anti-inflammatory factors in the adaptive immune response (Cobrin and Abreu, 2005; Targan and Karp, 2005; Zhang and Li, 2014). Interleukin (IL)-6, TNF-α, and IL-12(P40), which have generally been recognized as pro-inflammatory factors in DSS-induced colitis, are activated by antigen-presenting cells to perturb the balance of helper T cells and regulatory T cells (Wallace et al., 2014; Ramos and Papadakis, 2019). Elevated levels of IL-10, a cytokine with anti-inflammatory properties, have been found to be important for controlling intestinal inflammation (Saraiva and O'Garra, 2010; Wang et al., 2020). At the end of experimentation, our results showed that among the inflammatory groups, the BD-1 + Ceftri-DSS group had higher expression levels of IL-6, TNF-α, IL-12(P40), IFN-γ, and IL-17a mRNA in terms of local colonic immunity. However, in terms of systemic immunity, the Ceftri-DSS group had highest expression of TNF-α and IL-12(P40) mRNA in the spleen and IL-6 and TNF-α levels in sera among the inflammatory groups. Notably, BD-1 + Ceftri-DSS could better reduce the aforementioned indices compared to Ceftri-DSS and specifically elevate IL-10 mRNA expression in the inflammatory groups. Furthermore, immunization trends in each group on day 21 were similar but not as strong as that on day 46. In summary, early treatment with BD-1 alone had no significant effects on local and systemic immunity on days 21 and 46. Conversely, early intervention with BD-1 + Ceftri may activate adaptive immunity earlier or more strongly than other groups when colitis occurs in local immunity. However, the detailed and exact mechanism remains unclear and requires further exploration. Our results on the systemic immune system suggested that early treatment with ceftriaxone can promote a more active immune response among those with long-term colonic inflammation but not for those without inflammation. The above phenomenon is similar to the results of *in vitro* and animal experiment on the relationship between ceftriaxone and cytokine responses (Anuforom et al., 2016; Guo et al., 2017). The mechanism may be associated with the fact that ceftriaxone increases bacterial adhesion in the presence of infection, stimulating the production of immune cells but less so in the absence of bacteria (Anuforom et al., 2016). In this study, early intervention with BD-1 + Ceftri reduced the inflammatory stimulation caused by antibiotics to normal. Although the mechanism is still obscure, early treatment with BD-1 + Ceftri does not cause excessive immunity during inflammation and has a certain safety profile, which provides the basis for a new approach in controlling IBD.

Based on our results on immune changes in each group, this study further analyzed the gut microbiota to explore whether strains play a role by affecting the complex gut microbiota. IBD is a complex disease that involves interactions between genetic, environmental, and microbial factors in which intestinal microbes directly or indirectly affect the intestinal mucosal barrier and immune function through their metabolites (Ni et al., 2017; Glassner et al., 2020). Gut microbiota analysis on day 46 found that the NS-DSS group had a lower proportion of *Bacteroidetes/Firmicutes* and a higher relative abundance of *Proteobacteria* and *Escherichia/Shigella* compared to the NS-water group, which is consistent with the altered gut microbiota composition typical of IBD patients (Gophna et al., 2006; Frank et al., 2007; Stojanov et al., 2020). After early intervention with BD-1 and BD-1 + Ceftri, the relative abundances of *Escherichia/Shigella* and *Ruminococcus* were significantly lower at day 46 among the inflammation groups. Some studies have shown that the relative abundance of *Escherichia/Shigella* was specifically elevated in patients with IBD and that the low abundance of *Ruminococcus* was associated with low levels of inflammation (Henke et al., 2019; He et al., 2021; Kong et al., 2021). Conversely, both the BD-1-DSS and BD-1 + Ceftri-DSS groups an increased relative abundance of *Lactobacillus*, although the BD-1-DSS group had higher levels, whereas the relative abundance of *Bifidobacterium* was specifically elevated in the BD-1 + Ceftri-DSS group. For intestinal metabolites, the BD-1-DSS group maintained consistently higher concentrations of acetic acid, propionic acid, and butyric acid in both inflammatory and non-inflammatory states, where the production of SCFAs is a positive outcome for the relief of colitis symptoms (Sun et al., 2017; Goncalves et al., 2018). From these perspectives, the use of BD-1 + Ceftri in early life may increase the presence of *Bifidobacterium* the same species as BD-1 in the long-term gut. However, the use of BD-1 alone in early life may produce a forward cross-feeding effect through changes in metabolites of short-chain fatty acids, possibly resulting in increased *Lactobacillus* (Xiang et al., 2021). Immediately after combining these findings with our analysis of the gut microbiota after stopping treatment (on day 21), there were indeed differences in the composition of the gut microbiota after the intervention, which persisted until day 46. At day 21, ceftriaxone reduced the diversity of the microbiota and the content of SCFAs at the end of the intervention, with a decreased abundance of SCFAs-producing bacteria (such as, *Alistipeshe*, *Clostridium_XlVa*, and *Bifidobacterium*; Guo et al., 2017; Iyer and Corr, 2021). Early intervention with BD-1 does alter bacterial composition in terms of relative abundance, alpha diversity, and beta diversity. Interestingly, the relative abundance of *Bifidobacterium* showed a specific increase following early intervention with BD-1, although it did not persist until day 46. In contrast, the relative abundance of *Bifidobacterium* was not significantly increased after early treatment with BD-1 + Ceftri but did increase at day 46.

Based on the changes in the gut microbiota on days 21 and 46, we clearly found that although *Bifidobacterium* can colonize rather rapidly, they are easily covered by the original gut microbiota, and instead cross-feed *Lactobacillus* in the long-term. However, when antibiotics disrupt the host’s original microbiota structure, *Bifidobacterium* are more likely to survive longer. Therefore, we hypothesized that early intervention with BD-1 + Ceftri could alleviate the inflammatory symptoms of long-term colitis, which may be associated with the more prominent direct effects of the strain after antibiotic administration, such as specifically extending the survival time of some beneficial bacteria. Moreover, the long-term effects of direct BD-1 use may be associated with its regulation of gut microbiota and metabolites. Although more effects may come from metabolites, this remains uncertain. Therefore, the mechanism by which BD-1 promotes long-term anti-colitis action still remains unknown and needs further research.

Based on the above results, we found that the changes in gut microbiota and metabolites induced by appropriate intervention in early life persisted for some time. The direct stimulation of the intestinal barrier and immune response by probiotics in early life can also persist during colitis, which protected against inflammatory damage. On this basis, we speculate that gut microbiota and SCFAs (e.g., acetic acid and butyric acid) produced by gut microbiota from fermentation of dietary can directly or indirectly promote intestinal epithelial cell proliferation and goblet cell differentiation to promote intestinal development in early life. Similar findings were also described in the previous study (Koh et al., 2016). At the same time, after direct exposure to gut microbiota and SCFAs, intestinal epithelial cells can secrete a series of chemokines and cytokines, thereby activating innate and adaptive immune responses. Studies have also suggested that intestinal epithelial cells can activate potential immune cells to clear pathogens through innate signaling (such as Toll-like receptors and G-protein coupled receptors) pathways (Allaire et al., 2018). We were surprised in this study to find that gut microbiota-specific changes in early life can persist into long-term and trigger anti-inflammatory properties of colon tissue. Therefore, we believed that altering the gut microbiota during this special critical window period may be an important time window affecting the gut barrier and immune system, which may be related to the promotion of mucosal barrier immune maturation and the specific expression of gut development-related regulators (such as erythroid differentiation regulator-1; Al Nabhani et al., 2019; Abo et al., 2020; Lavelle and Sokol, 2020).

Overall, appropriate colonization of the gut microbiota in early life is essential to establishing the mucosal barrier and promoting immune maturation in early life and long-term. In the study, we find that the use of BD-1 + Ceftri in early life affected the construction of the gut microbiota and changes in local and systemic immunity and intestinal mucosal barrier. In particular, the changes induced by the BD-1 + Ceftri persisted for some time after stopping intervention and persisted during colitis, which protected against inflammatory damage. Therefore, we suspect that a more appropriate intervention in early life may be to disrupt the original host microbiota structure using antibiotics and then use a single probiotic strain, which may have a more prominent effect on composition of microbiota. This conjecture, if successful, would be similar to and more controllable than the FMT but would require further study to ascertain antibiotic dosages and timings, as well as probiotic strain selection. The current study found that although the direct use of a single strain in early life also has long-term efficacy in reducing colitis, the direct stimulation of the intestinal barrier and immune response by probiotics may be covered by the complex native gut microbiota. Thus, the anti-inflammatory effects of a single strain may be indirect, such as regulation of gut microbiota metabolites and cross-feeding with the original microbiota. Therefore, this experiment suggested that the colonization effects of the original microbiota cannot be ignored when using probiotics for early life intervention. Nonetheless, further research is needed on the interaction between single probiotics and the original microbiota, as well as the mechanism by which they affect health.

## Data availability statement

The datasets presented in this study can be found in online repositories. The names of the repository/repositories and accession number(s) can be found in the article/Supplementary material.

## Ethics statement

The animal study was reviewed and approved by Ethics Committee of West China Fourth Hospital and West China School of Public Health, Sichuan University.

## Author contributions

FH, XS, and CP designed the study. CP, JL, and ZM conducted the experiments. CP wrote the manuscript. CP, YuW, SWu, YiW, SWa, and RC performed the data analysis. FH, XS, RC, and CP revised the manuscript. All authors contributed to the article and approved the submitted version.

## Funding

This work was supported by the China Postdoctoral Science Foundation (2020M673267) and the Full-Time Postdoctoral Research and Development Fund of Sichuan University (2020SCU12010).

## Conflict of interest

The authors declare that the research was conducted in the absence of any commercial or financial relationships that could be construed as a potential conflict of interest.

## Publisher’s note

All claims expressed in this article are solely those of the authors and do not necessarily represent those of their affiliated organizations, or those of the publisher, the editors and the reviewers. Any product that may be evaluated in this article, or claim that may be made by its manufacturer, is not guaranteed or endorsed by the publisher.

## Supplementary materials

The Supplementary materials for this article can be found online at: https://www.frontiersin.org/articles/10.3389/fmicb.2022.916824/full#supplementary-material
