# Supplementary material for: Early life administration of Bifidobacterium bifidum BD-1 alleviates long-term colitis by remodeling the gut microbiota and promoting intestinal barrier development
Source: Front Microbiol. 2022 Jul 22;13:916824. doi: 10.3389/fmicb.2022.916824 (PMC9355606; doi:10.3389/fmicb.2022.916824)
Supplement: Supplementary file 1 [file Data_Sheet_1.pdf]

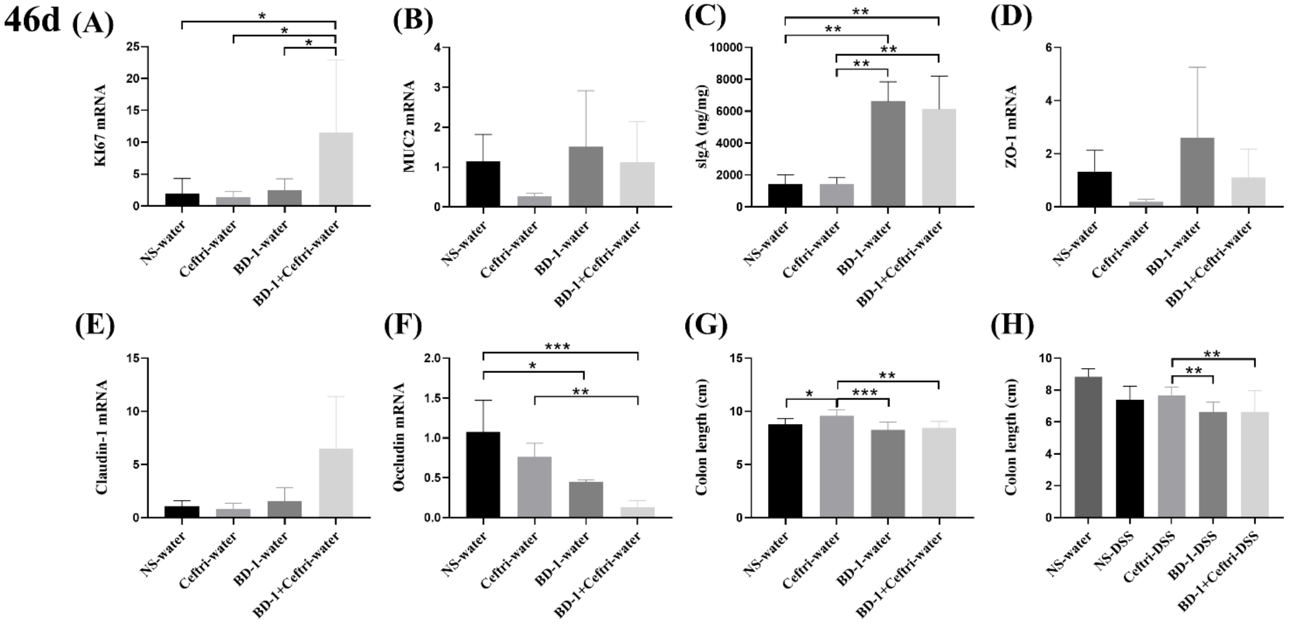

Figure S1. Intestinal development of mice (46d, n=6). (A) KI67 mRNA level. (B) MUC2 mRNA level. (C) Secretory Immunoglobulin A (sIgA) mRNA level in the cecum faeces. (D) ZO-1 mRNA level. (E) Claudin-1 mRNA level. (F) Occludin mRNA level. (G) Colon length (water groups). (H) Colon length (DSS groups). **P*<0.05, ***P*<0.01, ****P*<0.001 as conducted

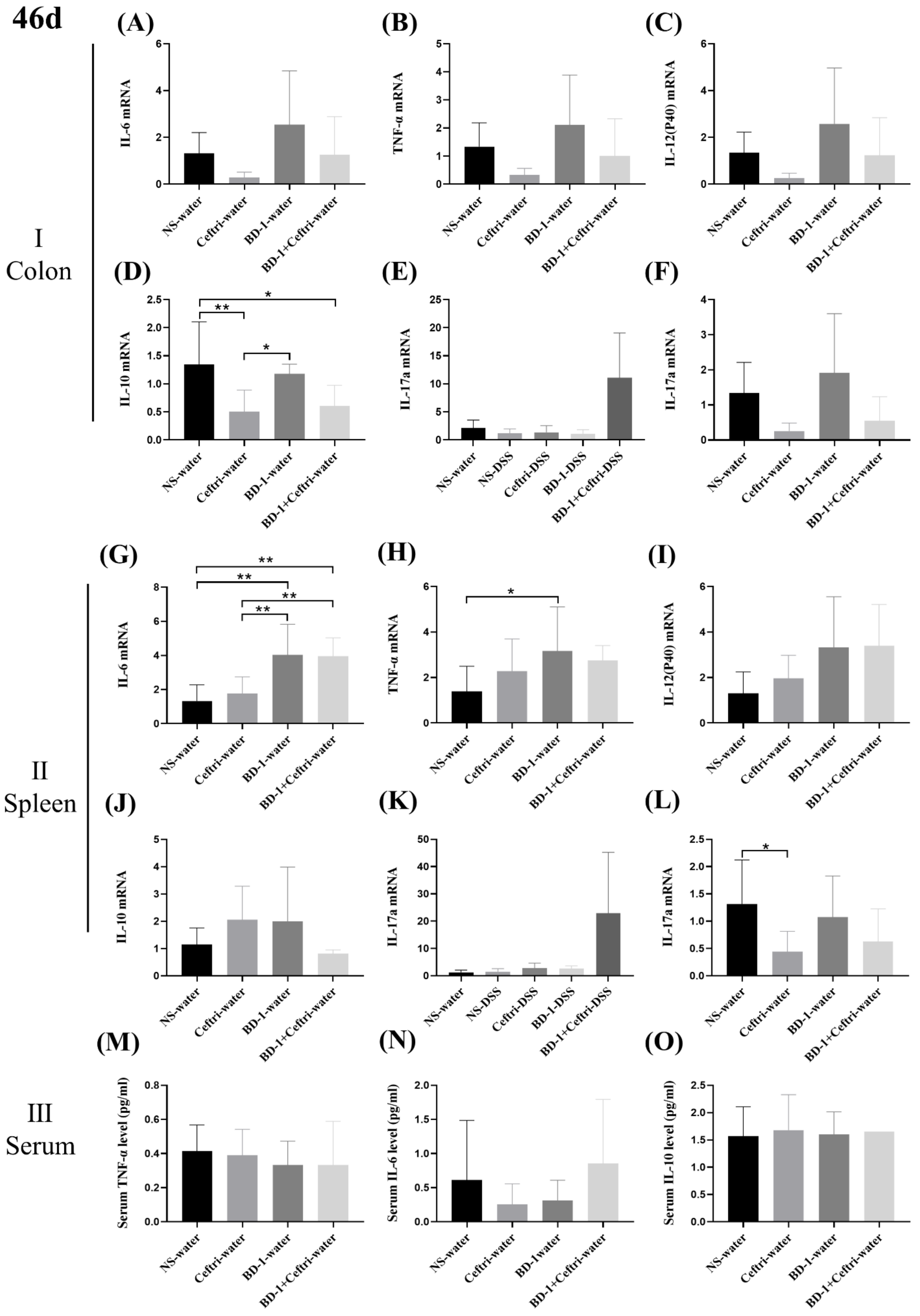
46d
I
Colon
II
Spleen
III
Serum
(A)
IL-6 mRNA
(B)
TNF-α mRNA
(C)
IL-12(P40) mRNA
(D)
IL-10 mRNA
(E)
IL-17a mRNA
(F)
IL-17a mRNA
(G)
IL-6 mRNA
(H)
TNF-α mRNA
(I)
IL-12(P40) mRNA
(J)
IL-10 mRNA
(K)
IL-17a mRNA
(L)
IL-17a mRNA
(M)
Serum TNF-α level (pg/ml)
(N)
Serum IL-6 level (pg/ml)
(O)
Serum IL-10 level (pg/ml)
NS-water
Ceftri-water
BD-1-water
BD-1+Ceftri-water
NS-DSS
Ceftri-DSS
BD-1-DSS
BD-1+Ceftri-DSS
BD-1water

Figure S2. Colonic local immunity of mice (46d, n=6). (A) Colonic IL-6 mRNA expression. (B) Colonic TNF-α mRNA expression. (C) Colonic IL-12(P40) mRNA expression. (D) Colonic IL-610 mRNA expression. (E) Colonic IL-17a mRNA expression (DSS groups). (F) Colonic IL-17a mRNA expression (water groups). (G) Splenic IL-6 mRNA expression. (H) Splenic TNF-α mRNA expression. (I) Splenic IL-12(P40) mRNA expression. (J) Splenic IL-10 mRNA expression. (K) Splenic IL-17a mRNA expression (DSS groups). (L) Splenic IL-17a mRNA expression (water groups). (M) Serum TNF-α level. (N) Serum IL-6 level. (O) Serum IL-10 level. **P*<0.05, ***P*<0.01, ****P*<0.001 as conducted.

46d

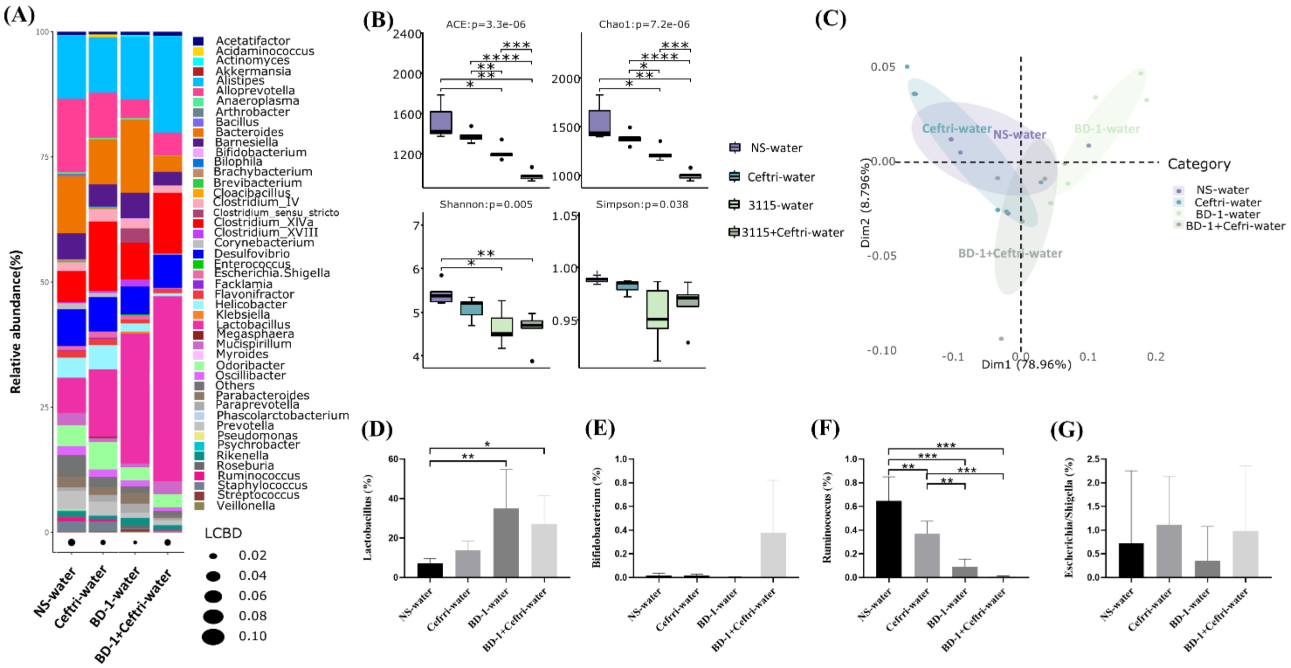

Figure S3. Gut microbiota in the non-inflammatory group (46d, n=5). (A) Relative abundance at the genus level. (B) The alpha diversity of the gut microbiota. (C) PCoA plots of fecal microbiota (Adnois, *P*<0.05; Betadisper, *P*>0.05). (D) Relative abundance of *Lactobacillus*. (E) Relative abundance of *Bifidobacterium*. (F) Relative abundance of *Ruminococcus*. (G) Relative abundance of *Escherichia/Shigella*. **P*<0.05, ***P*<0.01, ****P*<0.001, *****P*<0.0001 as conducted.

42d

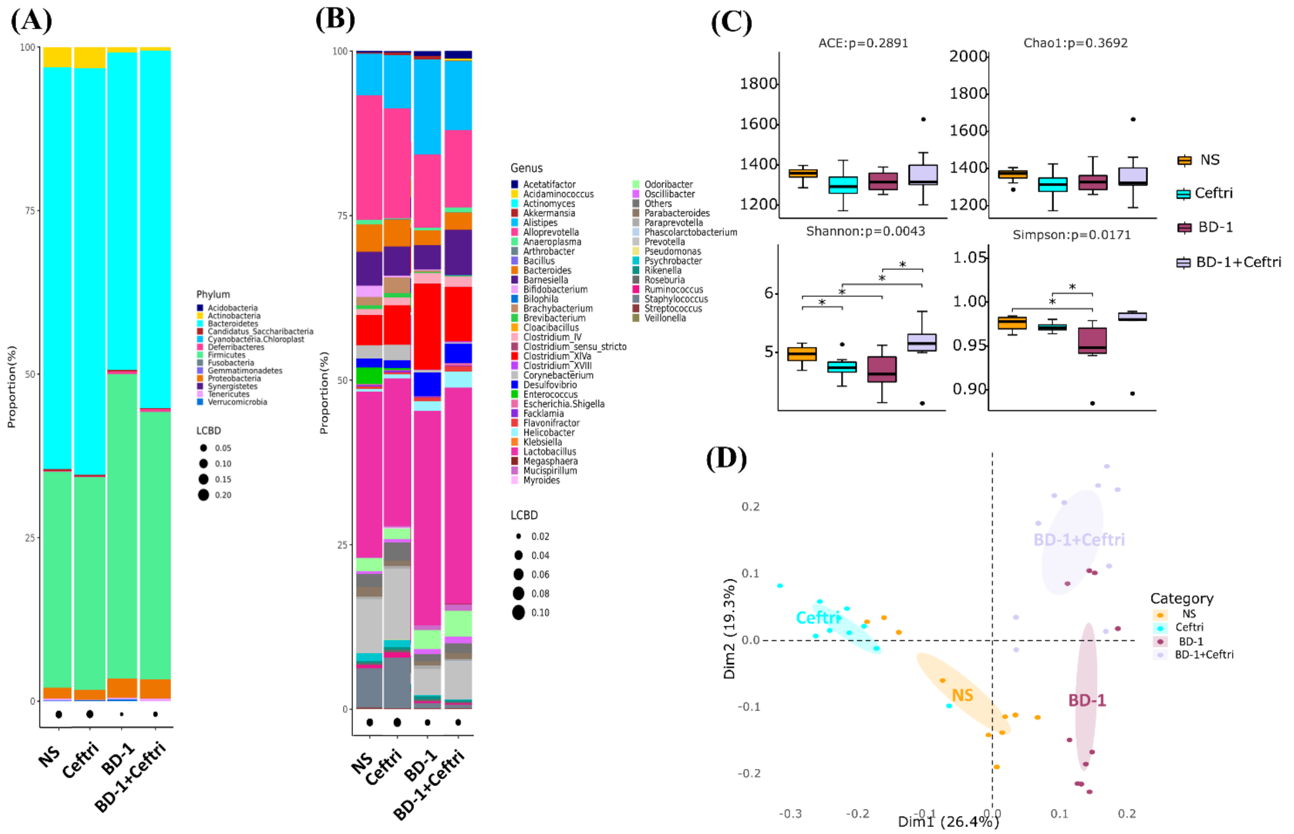

FigureS4. Changes in gut microbiota on day 42 (42d, n=5). (A) Relative abundance at the phylum level. (B) Relative abundance at the genus level. (C) The alpha diversity of the gut microbiota. (D) Principal co-ordinates analysis (PCoA) plots of fecal microbiota on weighted UniFrac distance (Adnois, $P<0.05$; Betadisper, $P>0.05$).

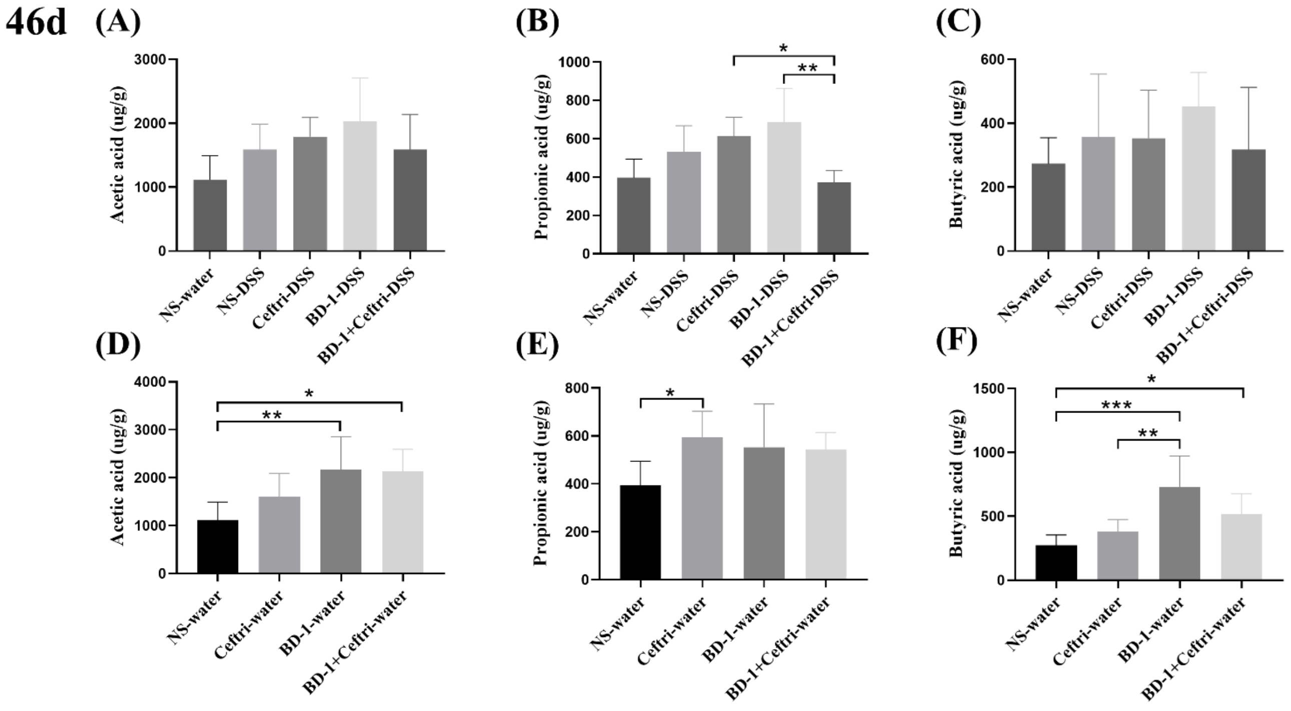

Figure S5. Intestinal metabolites of mice (46d, n=6). (A) Acetic acid level (DSS groups). (B) Propionic acid level (DSS groups). (C) Butyric acid level (DSS groups). (D) Acetic acid level (water groups). (E) Propionic acid level (water groups). (F) Butyric acid level (water groups). **P*<0.05, ***P*<0.01, ****P*<0.001 as conducted.
